# Identification of Differentially Expressed Genes through Integrated Study of Alzheimer’s Disease Affected Brain Regions

**DOI:** 10.1371/journal.pone.0152342

**Published:** 2016-04-06

**Authors:** Nisha Puthiyedth, Carlos Riveros, Regina Berretta, Pablo Moscato

**Affiliations:** 1 Information Based Medicine Program, Hunter Medical Research Institute, New Lambton Heights NSW, Australia; 2 Centre for Bioinformatics, Biomarker Discovery and Information-Based Medicine, School of Electrical Engineering and Computer Science, The University of Newcastle, Callaghan NSW, Australia; 3 Clinical Research Design, Information Technology and Statistics Suport Unit, Hunter Medical Research Institute, New Lambton Heights NSW, Australia; CNRS UMR7275, FRANCE

## Abstract

**Background:**

Alzheimer’s disease (AD) is the most common form of dementia in older adults that damages the brain and results in impaired memory, thinking and behaviour. The identification of differentially expressed genes and related pathways among affected brain regions can provide more information on the mechanisms of AD. In the past decade, several studies have reported many genes that are associated with AD. This wealth of information has become difficult to follow and interpret as most of the results are conflicting. In that case, it is worth doing an integrated study of multiple datasets that helps to increase the total number of samples and the statistical power in detecting biomarkers. In this study, we present an integrated analysis of five different brain region datasets and introduce new genes that warrant further investigation.

**Methods:**

The aim of our study is to apply a novel combinatorial optimisation based meta-analysis approach to identify differentially expressed genes that are associated to AD across brain regions. In this study, microarray gene expression data from 161 samples (74 non-demented controls, 87 AD) from the Entorhinal Cortex (EC), Hippocampus (HIP), Middle temporal gyrus (MTG), Posterior cingulate cortex (PC), Superior frontal gyrus (SFG) and visual cortex (VCX) brain regions were integrated and analysed using our method. The results are then compared to two popular meta-analysis methods, RankProd and GeneMeta, and to what can be obtained by analysing the individual datasets.

**Results:**

We find genes related with AD that are consistent with existing studies, and new candidate genes not previously related with AD. Our study confirms the up-regualtion of *INFAR2* and *PTMA* along with the down regulation of *GPHN, RAB2A, PSMD14* and *FGF*. Novel genes *PSMB2, WNK1, RPL15, SEMA4C, RWDD2A* and *LARGE* are found to be differentially expressed across all brain regions. Further investigation on these genes may provide new insights into the development of AD. In addition, we identified the presence of 23 non-coding features, including four miRNA precursors (miR-7, miR570, miR-1229 and miR-6821), dysregulated across the brain regions. Furthermore, we compared our results with two popular meta-analysis methods RankProd and GeneMeta to validate our findings and performed a sensitivity analysis by removing one dataset at a time to assess the robustness of our results. These new findings may provide new insights into the disease mechanisms and thus make a significant contribution in the near future towards understanding, prevention and cure of AD.

## Introduction

AD is a progressive and degenerative neurologic disorder characterised by the loss of mental ability. AD is the most common cause of dementia in older adults with loss of cognitive functions and memory. AD kills the nerve cells and makes changes in neurons and neurotransmitters that affect the communication between neurons and leads to brain function loss. The most common clinical features of AD are: the aggregation of β-amyloid into plaques, the presence of hyperphosphorylated Tau protein in self-assembled tangles and filaments, and the loss of connections between nerve cells in the brain that leads to brain atrophy [1]. Even though the process and development of AD is still unknown, it is likely that deterioration of the brain starts well before the problems become evident. The common symptoms of AD are difficulties in remembering recent events, thinking and reasoning, speaking and writing, making judgement and decisions, planning and performing familiar tasks and changes in personality and behaviour [2]. With the progression of AD, most parts of the brain get seriously damaged and shrinks dramatically due to widespread cell death. In advanced stage of AD, individuals lose their ability to communicate, to recognize family and loved ones and to care for themselves.

AD is not a part of normal aging, but increasing age is the strongest risk factor of AD. Three in ten people over the age of 85 and one in every eight people over 65 are estimated to develop AD. Family history and genetics, mild cognitive impairment (MCI), past head trauma, life style and heart health, life long learning and social engagement are the other risk factors of AD. The risk of developing AD is higher if a first degree relative (parent or siblings) has the disease. The genetic mechanism of AD among families remains unexplained. People with mild cognitive impairment have higher chance of developing AD, but is not a certainty and can be prevented by developing a healthy life style. Some studies show that the risk factors of heart disease may also increase the risk of developing AD [3, 4]. Studies also show the relationship between lifelong involvement in mentally and socially stimulating activities and reduced risk of AD [5, 6].

The diagnosis of AD is usually based on the patient’s medical history, mental status testing and physical testing. Even though several histopathological markers such as extracellular *β*-amyloid plaques and neurofibrillary tangles (NFTs) within neurons can determine AD presence [7], these can only be evaluated in the post-mortem brain or in rare surgical circumstances; physicians have then turned to other less invasive methods to diagnose AD, such as neuroimaging. Positron emission tomography (PET), which identifies the pattern of reduced glucose with the help of cerebral glucose metabolic rate [8], and magnetic resonance imaging (MRI), that identifies brain atrophy [9, 10], are two available imaging techniques for AD diagnosis. These techniques helps to find the damage in the tissue or vessels in the brain rather than predicting the risk of developing AD.

Most of the research studies of AD focus on the Hippocampus (HIP) brain region, as it is the first region to be affected by AD [11]. However, there are other regions that are functionally related with memory, attention, perceptual awareness, thought, language and consciousness—functions that are affected in AD. For example, the entorhinal cortex region (EC) works as a mediator for learning and memory. EC-HIP together play an important role in visual processing hierarchy and thereby receive signals for object representations [12]. The Posterior Cingulate cortex (PC) helps with visual perception and memory recollection [13]. The Middle Temporal Gyrus (MTG) is involved in some basic functions like recognition of faces, ascertaining of distance, etc. [14]. The Superior Frontal Gyrus (SFG) is associated with self-awareness and with the action of the sensory system [15]. The Visual Cortex (VCX) processes the visual information by receiving the visual data from the lateral geniculate body of the thalamus [16].

Since the disease symptoms characterisation in 1906 by Dr. Alois Alzheimer, genesis of AD has remained elusive. Only in 1993 the *APOE* gene was found to be associated to AD. Several studies have since been performed to identify other differentially expressed genes in AD affected brain regions [17]. However, ELISA measurement of β-amyloid, total Tau and Phospho-Tau-181 in cerebrospinal fluid (CSF) are the most advanced and accepted method for AD diagnosis. It is estimated that in 2050, approximately 80 million older people will suffer from AD [18]. Thus, it is of great challenge to find reliable biomarkers to understand the mechanism behind AD [19, 20].

An 18-protein signature in peripheral blood plasma was identified by Ray et al. [21] that can be used to predict the clinical symptoms of AD early before the symptoms are evident. They have used a single classifier approach to identify a panel of proteins that helps to decide whether patients with MCI will develop AD in the next 2–6 years. Soon after, Gomez-Ravetti et al. [22] used the same dataset and identified a five protein biomarker, subset of Ray’s 18-protein signature, that is sufficient to retrieve the same result with better accuracy to predict AD. Using Ray’s dataset, Rocha de Paula et al. [23] identified a specific pattern of cell signalling imbalance that can predict AD in patients with Mild Cognitive Impairment.

In 2010, Gómez Ravetti et al. [24] showed a clear pattern of up and down regulated genes related to the hippocampus region that reveals alterations in calcium, insulin, phosphatidylinositol and Wnt-signalling. They also found that the gene probes that are strongly correlated to AD severity are linked to synaptic function, neurofilament bundle assembly, and neuronal plasticity and inflammation. They showed that the gene homologous of *EGR1, zif268, Egr-1* or *ZENK*, together with other members of the *EGR* family, play an important role in short and long-term memory and neuronal plasticity in the brain. All these studies concentrated on a specific region data. A combined study of different brain regions may provide more information with regard of gene dysregulation driving the development and AD pathogenesis.

In 2008, Liang et al. [25] performed a combined study of postmortem gene expression data of six different brain regions and identified differentially expressed genes: *APOE, BACE1, STUB1 (CHIP), FYN, GGA1* and *SORL1*, also pinpointing genes with significant expression changes in AD across brain regions. [26] performed a four region study to gain knowledge about different regions and built a co-expression gene network to characterise the similarity and differences between the regions. They also found that the MTG region shows an early AD pathology compared to other regions. A network-based systems biology approach was proposed by Liu et al. [27] to study the AD related pathways and their dysfunctions among six brain regions. They identified the most significant AD related pathways across six brain regions.

Further, Lambert et al. [28] conducted a large scale two stage meta-analysis of genome wide association studies of 74,046 individuals. They identified 11 new susceptibility loci which are significant in relation to AD. Bertram et al. [29] performed a meta-analysis of AD genetic association studies and identified 20 polymorphisms in 13 genes that are closely related to AD. A Genome-Wide Association Meta-analysis of Neuropathologic Features of AD identified 9 new loci, involving the genes *ABCA7, BIN1, CASS4, CD33, MEF2C, MS4A6A, PICALM, SORL1* and *ZCWPW1*, that are significant in regards to AD pathogenesis [30].

Since the sample size of individual gene expression microarray datasets are small, computational methods can be used to integrate these gene expression data from different microarray studies. Greco et al. [31] proposed an integration method to combine microarray gene expression data from Affymetrix GeneChip experiments to investigate tissue selective expression patterns. A computational approach has been developed by Wang et al. [32] for genome-wide analysis of human tissue-selective gene expression data from heterogeneous sources.

In short, combined study of similarities and differences among different AD-affected brain region datasets can provide an better understanding of AD pathogenesis. Most of the studies report a large number of genes, and yet most of the results are conflicting [33]. Because of the exceedingly large number of AD related genes of different brain regions, it has become virtually impossible to systematically follow, evaluate, interpret or compare these findings.

A robust characterisation of the transcriptomic risk factors related to AD requires an integrated study. We performed a combined analysis using gene expression data from six different AD affected brain regions from the well-known Liang gene expression dataset [34]. The dataset contains data for the EC, HIP, MTG, PC, SFG and VCX regions and is re-analysed in this study to identify common genes that play an important role among six AD-affected brain regions. The regions considered in this study are briefly explained below.

Entorhinal Cortex(EC):
  EC is the main channel between HIP and the neocortex and is involved in the long-term cognitive memory formation [35]. In particular, EC supplies information to HIP from multiple senses and translates information to neocortex with the help of a neurotransmitter called glutamate. Studies have already been shown that EC is one of the region affected by AD in the early stage itself [36].
Hippocampus (HIP):
  HIP is a part of the temporal lobe that is absolutely necessary for forming new memories. It is common that AD affects the HIP early and severely before affecting any other part of the cortex [11], which shows memory is the first thing that starts to get falter in AD. Several studies shows that APOE plays a prominent role in HIP damage through impaired blood flow and the consequent lack of oxygen [37, 38].
The Middle Temporal Gyrus (MTG):
  MTG is a gyrus on the temporal lobe of the brain which is involved in a number of cognitive processes such as semantic memory, language processing and integration of information from different senses [14]. Many studies have shown the active neuronal loss for AD in the MTG region of the brain [39, 40].
The Posterior Cingulate Cortex (PC):
  PC is a part of the cingulate cortex which is highly connected and metabolically active brain region and functionally involved in learning and spatial memory. Studies have identified the amyloid deposition and reduced metabolism in PC in the progress of AD, also this region is significantly smaller in size in AD patients than controls [13, 41].
The Superior Frontal Gyrus (SFG):
  SFG is located at the superior part of the prefrontal cortex and it makes up about one third of the frontal lobe. Stimulation and activation of SFG is involved in self awareness and plays a role in working memory as well as manipulation of this memories to accomplish cognitive tasks like planning for the future, judgement, decision-making skills, attention span, and inhibition. Damage in SFG can cause in problems performing these functions [15, 42]. Several studies have been identified the presence of frontal hypo metabolism in relation with AD [43, 44].
The visual cortex (VCX):
  VCX is a part of cerebral cortex that occupies the entire surface of occipital lobe and functions as a visual data receiver. Damage of VCX can make the patient effectively blind even if their eyes are sending information from the visual field to VCX [45, 46]. Even though some studies shows changes in VCX related with normal aging, there is almost no information about changes in VCX in relation with AD [47].

We used the Coloured (α,β)-*k* Feature Set and Generalised (α,β)-*k* Feature Set approach [48] to conduct the combined study. As a robust feature selection method, the Coloured (α,β)-*k* Feature Set approach can handle the integrated dataset to find the minimum common set of genes that are significant to explain AD across regions. Also, we perform individual region analysis to identify the region specific genes and compare with the common genes. The functional and pathway analysis for the identified genes that are closely related with AD development is also performed.

The structure of the article is as follows; section Materials and Methods explains the datasets and present details of the methods employed in this study; the different signatures and data processing decisions are presented in Results. In Discussion, we analyse the most significant results.

## Materials and Methods

### Dataset and Pre-Processing

In this study we have used a publicly available Affymetrix microarray gene expression dataset for Alzheimer’s disease contributed by Liang et al.[34]. The dataset is deposited in Gene Expression Omnibus (GEO)[49] under the series number GSE5281. It contains 161 samples, 74 of which are non-demented controls and 87 affected with Alzheimer’s disease, with a mean age of 79.8 ± 9.1 years. The samples were collected (with a mean post-mortem interval of 2.5 hours) from three different AD centres from clinically and neuropathologically classified AD affected individuals. The samples were extracted from six different brain regions: EC, HIP, MTG, PC, SFG, VCX. The detail of samples in each region is given in Table 1.

**Table 1:** Sample details that belongs to different regions.

Region	Control	Affected	Total
EC	13	10	23
HIP	13	10	23
MTG	12	16	28
PC	13	9	22
SFG	11	23	34
VCX	12	19	31

**Region** is the name of different regions in the data: EC—Entorhinal Cortex, HIP—Hippocampus, MTG—Middle temporal gyrus, PC—Posterior cingulate cortex, SFG—Superior frontal gyrus, VCX—visual cortex. **Control** is the number of controls in each region. **Affected** is the number of diseased samples in the data. **Total** is the total number of samples in each region.

In this dataset, some samples in different regions correspond to the same individual; however this information is not available in the original study. One of the goals is to verify the robustness of the obtained signature accounting for inter-individual variability. We have used the accompanying clinical information to identify the samples across regions. As per the clinical data, there are overlapping and repeating samples in between regions. The details of the samples are given in S1 Table.

As a pre-processing step we applied Fayyad and Irani’s entropy-based heuristic on each region data to remove uninformative features. This is a univariate selection filter based on the Minimum Description Length Principle (MDL) [50]. The filter looks for (possibly a set of) discretisation threshold(s) maximising the class entropy gain. As different tissues have different gene expression profiles, the filter is applied to each region by separate. This method helps to remove the features that are not significantly different in control and AD samples and to reduce the dimensionality of the problem. It also facilitates the combinatorial approach by discretising the values of features.

### Individual Region Analysis

On each region we applied (α,β)-*k* Feature Set approach to obtain a region-specific signature. This approach provides a significant set of genes that collectively maximise the inter-class discrimination and the intra-class coherency [51, 52]. The method helps to select a minimum set of features that collectively provide a maximum amount of evidence to differentiate the control and AD samples in each brain region. The resulting probes are annotated using BioMart [53].

### Combined Analysis

For the integrated analysis of the selected datasets we have used Coloured (α,β)-*k* Feature Set approach and compared our results with two other popular meta-analysis methods: RankProd [54] and GeneMeta [55]. RankProd is a non-parametric meta-analysis tool that use Fold Change (FC) of each gene to rank and compare genes within each region. GeneMeta is an R package based on the meta-analysis method proposed by Choi et al. [56] in which an overall ranked gene list is produced based on the False Discovery Rate (FDR) of each gene.

#### Coloured (αβ)-*k* Feature Set Approach

We have recently proposed a combinatorial optimisation based method called Coloured (α,β)-*k* Feature Set approach [48] that can handle the integration of datasets in a consistent manner and selects the minimum set of significant features that can differentiate sample pairs across multiple datasets.

The decision versions of the Coloured and Generalised (α,β)-*k* Feature Set problems are given in [48] and reproduced below for convenience. Let B represent a set of binary values, i.e. B={0,1}; let *n* be the number of features and *m* the number of samples, *p* be the number of sample groups (i.e. different platforms/cohorts/datasets) and the tuple *y* be the class labels of the samples.

**Coloured (a,b)-*k* Feature Set:**
*Instance:* A set $X=\{x_{i}|x_{i}\in B^{n}\wedge 1\leq i\leq m\}$, a colouring function *c*: {1, …, *m*}→{1, …, *p*}, a tuple $y\in B^{m}$, integers *α* > 0, *β* ≥ 0, *k* > 0.
*Parameter:* *α*, *β* and *k*.
*Question:* Is there a set *I* ⊆ {1, …, *n*} with |*I*|≤*k* such that for all *i*, *j* ∈ {1, …, *m*} where *c*(*i*) = *c*(*j*)
  - if *y*_*i*_ ≠ *y*_*j*_ there exists $I_{i,j}^{\alpha }\subseteq I$ with $|I_{i,j}^{\alpha }|\geq \alpha$ such that *x*_*i*, *s*_ ≠ *x*_*j*, *s*_ for all $s\in I_{i,j}^{\alpha }$,
  - if *y*_*i*_ = *y*_*j*_ there exists $I_{i,j}^{\beta }\subseteq I$ with $|I_{i,j}^{\beta }|\geq \beta$ such that *x*_*i*, *s*_ = *x*_*j*, *s*_ for all $s\in I_{i,j}^{\beta }$?

In words, the Coloured (α,β)-*k* Feature Set problem instance is constructed from a collection of individual (α,β)-*k* Feature Set instances with common features, where the comparison of feature values is limited to sample pairs formed from each individual instance. The “coloured” name stems from assuming samples in each individual instance are coloured with the same unique colour, then only same coloured samples can be combined in pairs.

**Generalised (a,b)-*k* Feature Set:**
*Instance:* A set $X=\{x_{i}|x_{i}\in B^{n}\wedge 1\leq i\leq m\}$, a function g:{1,…,m}×{1,…,m}→B, a tuple $y\in B^{m}$, integers *α* > 0, *β* ≥ 0, *k* > 0.
*Parameter:* *α*, *β* and *k*.
*Question:* Is there a set *I* ⊆ {1, …, *n*} with |*I*|≤*k* such that for all *i*, *j* ∈ {1, …, *m*} where *g*(*i*, *j*) = 1
  - if *y*_*i*_ ≠ *y*_*j*_ there exists $I_{i,j}^{\alpha }\subseteq I$ with $|I_{i,j}^{\alpha }|\geq \alpha$ such that *x*_*i*, *s*_ ≠ *x*_*j*, *s*_ for all $s\in I_{i,j}^{\alpha }$,
  - if *y*_*i*_ = *y*_*j*_ there exists $I_{i,j}^{\beta }\subseteq I$ with $|I_{i,j}^{\beta }|\geq \beta$ such that *x*_*i*, *s*_ = *x*_*j*, *s*_ for all $s\in I_{i,j}^{\beta }$?

The Generalised (α,β)-*k* Feature Set problem has been devised to deal with the more general situation in which some samples in one sample group may be compared to samples in another sample group, for example. The binary function *g* indicates when feature values for a given arbitrary sample pair can be compared.

Even though the samples have been presented as an array of binary values, it is not strictly necessary. The class label can be a categorical variable taking values over a (typically small) set of categories or classes. The features can have values of any kind, as long as there exists a meaningful comparison able to decide if any two values are considered as significantly different or not. The entropy filtering and discretisation helps the comparison by providing an information theory-based objective criterion of what the discretisation thresholds are, while being able to support multi-modal value distributions.

In our case, the different datasets are each brain region data. For this, we selected the probes from all the six regions data that pass the Fayyad and Irani’s entropy-based heuristic test. The combined region data is prepared according to the selected probes by combining the specific discrete values of those probes from each region. The Coloured (α,β)-*k* Feature Set approach has been applied on the combined region data. The approach provided us with a set of probes that are differentially expressed in all the regions. The resulting probes are annotated using BioMart [53].

### Functional and Pathway Analysis

The pathway analysis has been performed using the Expression Analysis Systematic Explorer (EASE) [57] to obtain the EASE score, which is a modified version of Fisher Exact *p*-value used for gene-enrichment analysis, to identify the dysregulated pathways. EASE score < 0.06 represents that the gene is specifically related with the pathway in the context of the provided list.

To simplify the functional and pathway analysis, we applied Bonferroni correction, which is a conservative adjustment to the EASE score in order to control the multiple comparison effect, on the resulting list of probes from individual and combined analysis and selected the probes that have a Bonferroni corrected *p*-value (BF-value) < 0.0001. Also, we have selected the top 15 probes according to the BF-value to discuss further in relation with AD.

The resulted list of genes have been compared with the list of genes expressed in the normal brain collected from The Human Protein Atlas [58] and explained in the Discussion.

#### Other Meta-Analysis Methods

As comparison benchmark we have used two other widely popular meta-analysis methods. RankProd is a non-parametric meta-analysis tool introduced by Hong et al. [54] to identify differentially expressed genes from the integrated dataset. Fold Change (FC) of each gene is used to rank and compare genes within each region. An overall ranked gene list is produced by aggregating the individual ranks across regions as,

$$\begin{array}{c} RP_{g}={\left(\prod_{i}^{K}r_{gi}\right)}^{1/K} \end{array}$$

where *RP*_*g*_ is the overall rank product of gene *g*, which is the product of rank ratio *r*_*gi*_ of each comparison with a total of *K* comparisons.

In this study, we combined all the six region data without any pre-processing as the data belongs to the same platform. We have then applied RankProd on the combined region data to select genes that can discriminate the control and AD samples.

A meta-analysis method designed for same platform situations is GeneMeta. It was introduced by Lusa et al. [55] as an R package based on the meta-analysis method proposed by Choi et al. [56]. An overall ranked gene list is produced based on the False Discovery Rate (FDR) of each gene. FDR is calculated as,

$$\begin{array}{c} FDR=\frac{\frac{1}{B}\sum_{b}\sum_{j}I\left(\left|Z_{j}^{*b}\right|\geq z_{th}\right)}{\sum_{j}I\left(\left|z_{j}\right|\geq z_{th}\right)} \end{array}$$

where *B* is the number of column wise permutations performed in each dataset, each of them is represented as *b* = 1, 2, …, *B*. *Z*_*j*_ is the average effect size for gene *j*. The total number of datasets are denoted as *I*, where *I*(⋅) is the indicator function (equals to 1 if the condition in parenthesis is true and 0 otherwise). The denominator represents the number of genes that are significant in the data, and the numerator is the expected number of falsely significant genes. In this study, we combined all regions data without any pre-processing and applied GeneMeta to get the genes that are differentially expressed in the combined region data.

#### Sensitivity Analysis

We have analysed the robustness of the final integration results with respect to varying compositions of the individual region data. We have repeated the above mentioned steps with different combination of region data prepared by removing single or multiple regions from the combined data. This step helps to identify the most significant genes which are not dependent on a single region, as well as each region’s contribution to the final results. Specifically, we performed the following steps: a) removal of the region EC from the combined dataset b) removal of the region HIP from the combined datasets, and (c) removal of the regions MTG and PC from the combined dataset. Then obtained the list of probes that can distinguish the classes by applying the Coloured (α,β)-*k* Feature Set approach in each case obtained and compared with our original result.

## Results

To address region-specific vulnerability with AD pathology and complexity, we performed a comparative study of six different regions (EC, HIP, MTG, PC, SFG, VCX) of brain of individuals with AD and non demented controls using Affymetrix Human Genome U133 Plus microarrays. The purpose of our study is to identify significant genes in each region associated with the presence of AD, to identify common genes among all the regions, and to bring together all these results with previous studies to sketch a region specificity in relation to AD.

### Individual Analysis Results

We identified the probes that are differentially expressed by applying (α,β)-*k* Feature Set approach [51, 52] on each region separately. Differentially expressed genes and specific dysregulated pathways together provide new insights to the pathogenesis of AD. The differentially expressed genes and related pathways for each region are analysed and explained in the following sections.

The analysis of each region results in a long list of genes that are significantly related with AD. The list of genes related with each region is given in S2 Table. For functional and pathway analysis, we have selected the genes that have Bonferroni-corrected *p*-value (BF-value) < 0.0001. The number of resulting genes before and after applying Bonferroni correction for each region is given in Table 2. The list of pathways and the related details for each region is given in S3 Table. A gene ordering algorithm introduced by Moscato et al. [59] has been applied on the resultant set of genes for each region to generate a heatmap that brings out the correlation between the genes. The heatmaps for all the regions with differentially expressed genes are given in S5 Table. We also find some probes corresponding to microRNA precursors in each region and are given in S6 Table. We must notice that the gene expression microarray platform used in this study, Affymetrix HGU133 plus v2.0, is only capable of detecting microRNA precursors and not mature microRNA sequences. The mention of these precursors is however relevant, as they are a necessary for the synthesis of functional mature sequences.

**Table 2:** Summary of Significant genes in different brain regions.

Region	Probes	Result	Genes	BF_signif_	Pathways	microRNAs
**EC**	11504	4558	3762	108	24	10
**HIP**	11501	7779	5594	475	55	12
**MTG**	12607	6398	4941	1138	81	13
**PC**	15907	12690	7402	206	21	22
**SFG**	8785	5473	4344	47	23	13
**VCX**	5332	2185	1900	11	2	3

**Region** is the acronym of the different brain regions in the data. **Probes** is the number of probes before applying (α,β)-*k* Feature Set approach. **Result** is the number of probes resulted from the (α,β)-*k* Feature Set approach for each region. **Genes** is the number of genes obtained by annotating the resultant signature. BF_signif_ is the number of genes that are used for further analysis with a Bonferroni corrected *p*-value < 0.0001. **Pathways** is the number of related pathways by annotating the genes with the BF_signif_ genes. **microRNAs** is the number of resulted microRNA precursors for each region.

#### Common Probes in Individual Region Signatures

Studies reported that VCX is a less metabolically affected region and shows the least amount of AD related genes and changes [60, 61, 62]. As per our individual region analysis result, VCX shows less number of common genes with other regions. In spite of the large size of individual region signatures, there are only 67 common genes among the individual signatures. The VCX region does not show a large overlap with other regions; removal of the region signture increase the number of common genes to 288 among the five other region signatures. Based on this, we have excluded the region VCX from our combined analysis.

### Combined Analysis Results

#### Coloured (α,β)-*k* Feature Set Approach Results

**Fig 1:**
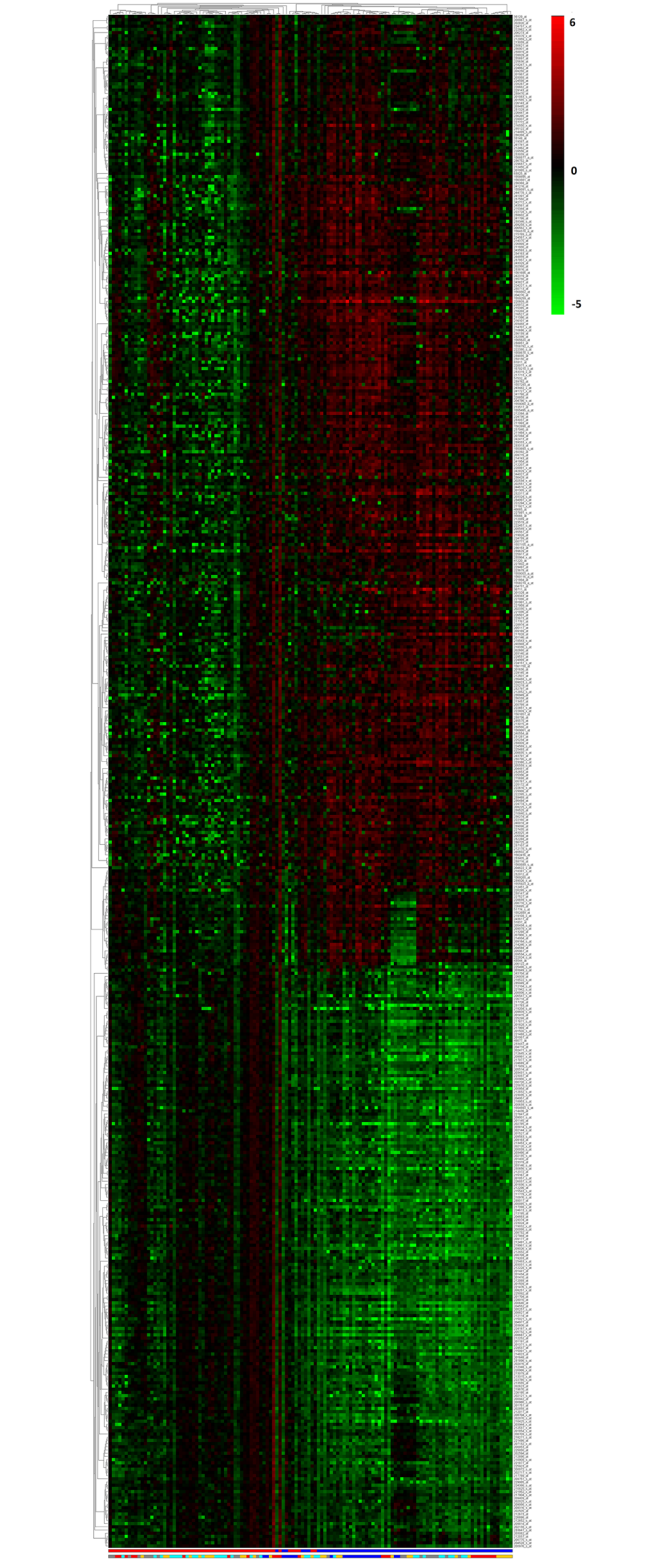
Heatmap for the 479 probes with BF-value < 0.0001 of combined analysis. There are 479 up and down regulated probes which are differentially expressed between control and AD. The first colour bar at the bottom indicates AD (blue) and control (red) samples. The second colour bar represents each sample group in different colour. EC (blue), HIP (red), MTG (orange), PC (grey) and SFG (cyan). The colour bar at the side of the heatmap represents the range of fold-changes with respect to the control samples mean value by means of a colour gradient ranging from green (log_2_(*FC*) = −5, down regulation) to red (log_2_(*FC*) = 6, up-regulation). See S1 Fig for a full size version of this figure.

To perform the combined analysis, we prepared a combined dataset by integrating all the five regions (EC, HIP, MTG, PC and SFG), selecting the probes that pass Fayyad and Irani’s entropy-based heuristic test (as explained in Coloured (αβ)-*k* Feature Set Approach). The combined dataset contains 3120 features and 126 samples. We have performed the combined analysis in two ways as different region data shares same samples. First, we applied Coloured (α,β)-*k* Feature Set approach (refer to Coloured (αβ)-*k* Feature Set Approach) on the combined dataset and obtained a list of 825 differentially expressed probes with maximum *α* and *β* value of 396 and 300 respectively, which is annotated to 728 genes. In this list, 479 have a BF-value < 0.0001, mapping to 67 significant pathways with EASE score < 0.06. The list of resulted probes with related details and pathways are given in S7 Table. The heatmap for 479 probes are given in Fig 1.

**Fig 2:**
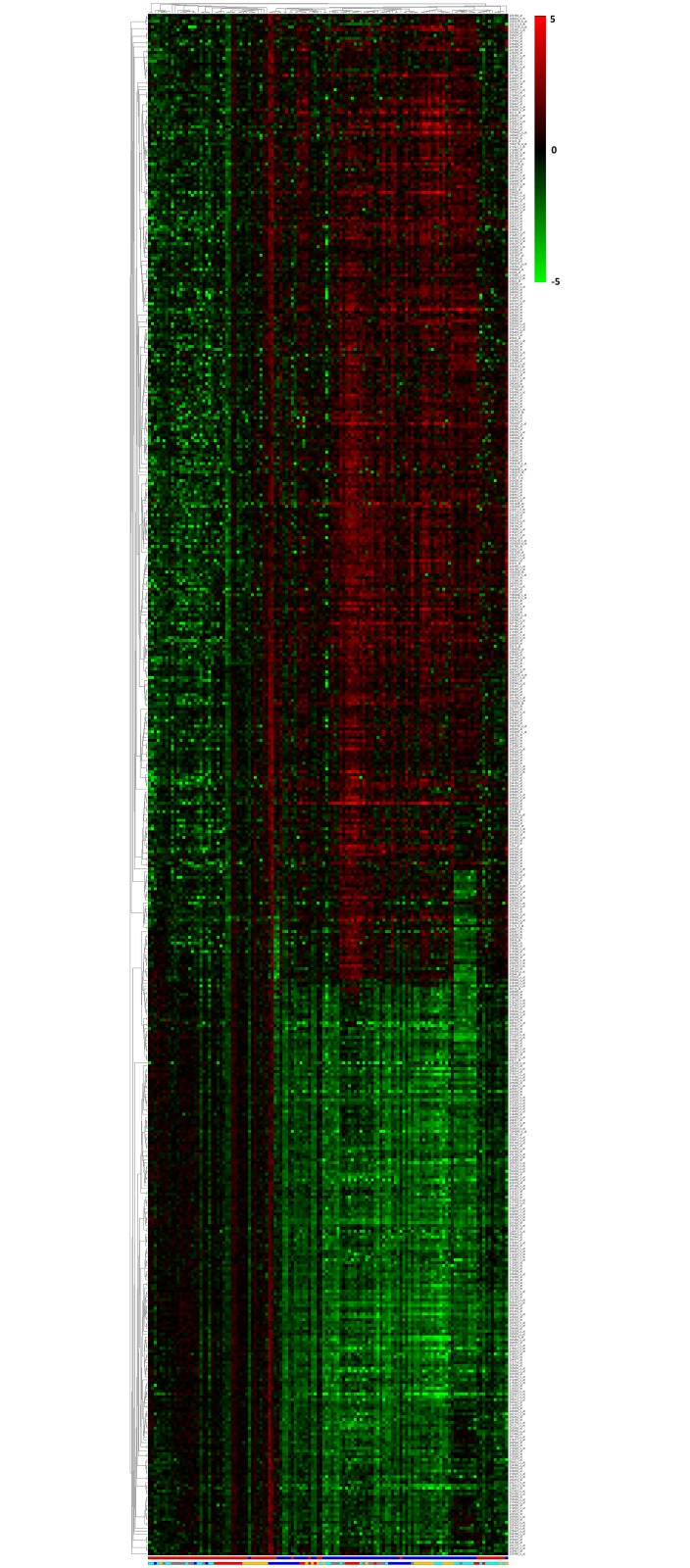
Heatmap for the 540 probes with BF-value < 0.0001 of combined analysis. There are 540 up and down regulated probes which are differentially expressed between control and AD. The first colour bar at the bottom indicates AD (blue) and control (red) samples. The second colour bar represents each sample group in different colour. EC (blue), HIP (red), MTG (orange), PC (grey) and SFG (cyan). The colour bar at the side of the heatmap represents the range of fold-changes with respect to the control samples mean value by means of a colour gradient ranging from green (log_2_(*FC*) = −5, down regulation) to red (log_2_(*FC*) = 5, up-regulation). See S2 Fig for a full size version of this figure.

In the next step, we applied the Generalised (α,β)-*k* Feature Set approach on the combined dataset to test whether correlation between samples in different regions might provide a more robust disease signature (refer to Coloured (α,β)-*k* Feature Set Approach for more details); we obtained a list of 871 differentially expressed probes with maximum *α* and *β* values of 396 and 311 respectively, which is annotated to 747 genes. In this list, 540 have a BF-value < 0.0001, mapping to 70 significant pathways that have EASE score < 0.06. The list of probes with related details and pathways are given in S7 Table. The heatmap for 540 probes are given in Fig 2. The Coloured (α,β)-*k* Feature Set and Generalised (α,β)-*k* Feature Set have 473 genes that are common between them, that shows a high level of agreement between the results. From the increased *β* value we deduce that the association of samples across different regions provided us a slight increase in the intra-class coherency of the description.

**Fig 3:**
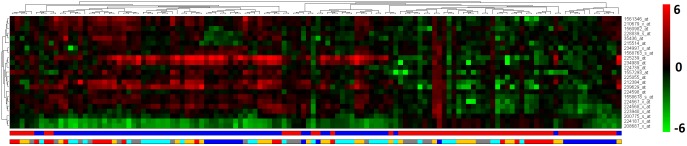
Heatmap for the 23 probes from the combined analysis result that annotate to the non coding features. There are 23 up and down regulated probes which are differentially expressed between control and AD. The first colour bar at the bottom indicates AD (blue) and control (red) samples. The second colour bar represents each sample group in different colour. EC (blue), HIP (red), MTG (orange), PC (grey) and SFG (cyan). The colour bar at the side of the heatmap represents the range of fold-changes with respect to the control samples mean value by means of a colour gradient ranging from green (log_2_(*FC*) = −6, down regulation) to red (log_2_(*FC*) = 6, up-regulation).

From the resulting list of probes from Coloured (α,β)-*k* Feature Set approach, we find 23 non coding features differentially expressed across EC, HIP, MTG, PC and SFG regions. The pathway analysis of these non coding features resulted with 13 pathways given in Table 3. These include four microRNAs: hsa-mir-7-1, hsa-mir-570, hsa-mir-1229 and hsa-mir-6821. The details of microRNAs are later discussed in Discussion. A heatmap for the 23 features is given in Fig 3.

**Table 3:** Dysregulated pathway related to non coding features.

Probe ID	Gene Symbol	Gene Name	Pathway
239629_at	*RNU7-45P*	RNA, U7 small nuclear 45 pseudogene	Apoptosis, FAS signalling pathway
208687_x_at, 224187_x_at	*SNORD14E; MALAT1*	small nucleolar RNA, C/D box 14E; metastasis associated lung adenocarcinoma transcript 1	Spliceosome, MAPK signaling pathway, Endocytosis, Antigen processing and presentation, parkinson disease, Membrane Trafficking
200775_s_at	*MIR7-1*	microRNA 7-1	Spliceosome, Processing of Capped Intron-Containing Pre-mRNA, Influenza Infection, Gene Expression,
224598_at	*MIR1229*	microRNA 1229	N-Glycan biosynthesis,
1560982_at	*CNKSR3*	CNKSR Family Member 3	Tight junction, Signalling by GPCR

**Probe ID** is the probe id related to the respective non coding features.**Gene Symbol and Name** is the associated gene in relation to the non coding feature that is involved in the pathway.**Pathway** is the name of the pathway.

The comparison between Coloured (α,β)-*k* Feature Set approach result with the list of brain related genes from Human Protein Atlas shows 328 genes from our result that are normally expressed in human brain are dysregulated in AD. Also we identified an additional 17 genes which are not expressed in normal human brain but appear over expressed in AD. The average log_2_ of fold change for the over and under expressed genes in AD are 5 and 4.5 respectively with respect to the control samples mean value, across all regions.

#### RankProd Results

We have used the well known meta-analysis RankProd method to have a comparison of our resulting list of genes. The application of RankProd on the combined dataset has ordered the list of genes by increasing percentage of false positive likelihood value and selected the top up and down regulated genes with 0.05 cut-off. This resulted in a list of 6908 up regulated genes and 5853 down regulated genes. The comparison of (α,β)-*k* Feature Set approach result with RankProd result shows that 760 out of our 825 probes are present in the top list of RankProd. Also the comparison of Generalised Coloured (α,β)-*k* Feature Set approach result with RankProd result shows 802 out of 871 probes are present in the result of RankProd. That shows a high level of agreement between the results. The resulting probes from RankProd and the comparison with our result is given in S8 Table.

#### GeneMeta Results

We also compared our result with another well known meta-analysis method GeneMeta, in which the probes are ordered based on the FDR. Application of this method on the combined dataset resulting with 17964 probes with a FDR cut-off of 0.05, that is recommended in the method. The comparison of Coloured (α,β)-*k* Feature Set approach result with GeneMeta result shows that 684 out of 825 probes are present in the top list of GeneMeta. Also the comparison of Generalised (α,β)-*k* Feature Set approach result with GeneMeta result shows 742 out of 871 probes are present in the result of GeneMeta. That shows a high level of agreement between the results. The GeneMeta resulting probes and the comparison with our result is given in S8 Table.

#### Sensitivity Analysis Results

We performed a sensitivity analysis by removing one or more datasets at a time and compared the result with the original Coloured (α,β)-*k* Feature Set approach result. The results of all three cases shows between 33% to nearly 50% agreement with the original result, and more importantly, more than 90% of probes with BF-value < 10^-5^. This shows the robustness of the combined analysis results in the presence of large variations, such as the removal of on or more datasets. The list of genes resulting when removing different regions are given in S9 Table.

## Discussion

As AD progresses, Tau pathology starts spreading from one brain region to another, first in EC, next in HIP, and then cerebral cortex, in a consistent pattern. These brain regions are interconnected through synapses that create communication networks [63]. Studies have demonstrated that AD is closely related with the alterations in the connectivity between different brain regions [64]. Although studies of gene expression changes in different brain regions in relation with AD has been performed separately, a combined study to understand the overlap and difference between different brain regions has been lacking. Here we analysed the differential expression of genes through a combined study of five different brain regions.

Our study help us identify the set of genes and related pathways that may play important role in the development of AD. The individual analysis of each region, EC, HIP, MTG, PC, SFG and VCX, provided a set of genes and pathways that are highly significant with that region alone. The set of genes highly associated with all the regions is identified by the combined analysis of EC, HIP, MTG, PC and SFG. As mentioned before the region VCX has been eliminated from the combined study since individual results have little in common with the other regions and its inclusion restricts the amount of common evidence that can be obtained from the integrated analysis. Only two pathways and 67 genes appear as significant when common genes of region-specific analysis results of EC, HIP, MTG, PC and SFG are annotated. The common pathways are: Metabolism of cofactors and vitamins and sorting and degradation. However, the combined analysis using Coloured (α,β)-*k* Feature Set approach produces 62 dysregulated pathways across the five different regions. Our common signature is of considerable reduced size as compared to rank- and statistically-based meta analysis methods. At the same time, the comparison shows our results in high level of agreement with other methods, while the sensitivity analysis provides evidence of the robustness and ability of the method to capture a large number of the most significant results, even with a relatively low number of samples. This gives us confidence to proceed to the analysis of the top 10 altered pathways and genes, listed in Table 4.

**Table 4:** Top dysregulated pathways.

Pathway	Gene Count	Gene Symbol
Carbohydrate Metabolism	12	*ACACB; ALDOA; GRHPR; IDH3A; IDH3B; IDH3G; ME3; PDHA1; PFKFB3; PFKM; PRPS1; TPI1;ABAT; AKR1B1*
Lipid Metabolism	4	*ACAA1; ACACB; HSD17B7; NQO2*
Alzheimer’s Disease	3	*LPL; SNCA; GNG3*
Citrate Cycle (TCA Cycle)	3	*IDH3A; IDH3B; IDH3G*
ATP Synthesis	6	*ATP5B; ATP5G1; ATP5J2; ATP6V0B; ATP6V1E1; ATP6V1F*
Cell Communication	4	*CAPNS1; SORBS1; TLN2; CAV2*
Pentose Phosphate Pathway	3	*ALDOA; PFKM; PRPS1*
Amino Acid Metabolism	8	*ABAT; ACAA1; ADSL; EPRS; GFPT1; GOT2; GSS; PDHA1*
Signal Transduction	11	*DGKG; EGFR; INPP4A; ITPR2; PRKCA; PRKCE; PRKCZ; PTPN2; PTPN3; PTPRD; PTPRM*
Purine metabolism	8	*ADCY1; ADSL; AK3; ENTPD6; GUCY1A3; IMPDH2; NME7; PRPS1*

**Pathway** is the pathway name.**Gene Count** is the number of genes from our result that is involved in the pathway.**Gene Symbol** are the gene symbols.

The pathways are mainly related to the classes of Carbohydrate metabolism, Amino acid metabolism, Signal transduction and lipid metabolism. Carbohydrates are the important source of energy that maintain the life of living cells. The carbohydrate metabolic pathways have been previously implicated in relation with AD progression. Henderson [65] shows that consumption of high carbohydrate diet may be a cause of the primary event that leads to the development of AD [65]. A series of studies suggest that relatively simple preventative measures like lower consumption of starchy carbohydrates and high essential fatty acids in the diet may effectively prevent AD [65–69]. Moreover, studies shows that carbohydrate diets can lead to dysreguration of LPL (Lipoprotein Lipases) activity and increase insulin sensitivity [65]. Our study also shows the under expression of the LPL gene that participate in the AD pathway. The increased activity of Glycolysis, galactose metabolism [70, 71] and pentose phosphate pathway [72, 73] has already been associated with increased AD risk. All these studies indicate that the risk for Alzheimer’s disease can be reduced with a balanced diet of protein, carbohydrate and fat.

Amino acids are the building blocks of proteins and the dysregulation of amino acid processing can result from defects either in the breakdown of amino acids or in the transport of amino acids into cells. The over representation of amino acid metabolism has also been reported to be associated with AD [74, 75].

Lipids plays a major role in the cell signalling especially in brain and are the major energy reserve in the brain cells and tissues. Studies shows that the abnormal lipid metabolism contributes to the pathogenesis of AD and other neurodegenerative disorders [76, 77]. According to the literature search, all the resulting pathways are closely related with the development of AD.

Next, we discuss about the top 15 differentially expressed genes across all the five regions from the result of Coloured (α,β)-*k* Feature Set approach, given in Table 5.

**Table 5:** The top most differentially expressed genes across EC, HIP, MTG, PC and SFG.

Probe ID	Gene Symbol	Gene Name	Location
210976_s_at	*PFKM*	Phosphofructokinase, muscle	12q13.11
200039_s_at	*PSMB2*	Proteasome (prosome, macropain) subunit, beta type, 2	1p34.2
211993_at	*WNK1*	WNK lysine deficient protein kinase 1	12p13.3
221476_s_at	*RPL15*	Ribosomal protein L15	3p24.1
211921_x_at	*PTMA*	Prothymosin, alpha	2q37.1
46665_at	*SEMA4C*	Sema domain, immunoglobulin domain (Ig), transmembrane domain (TM) and short cytoplasmic domain, (semaphorin) 4C	2q11.2
223319_at	*GPHN*	Gephyrin	14q23.3
208732_at	*RAB2A*	RAB2A, member RAS oncogene family	8q12.1
213555_at	*RWDD2A*	RWD domain containing 2A	6q15
203146_s_at	*GABBR1*	Gamma-aminobutyric acid (GABA) B receptor, 1	6p21.3
224567_x_at	*MALAT1*	Metastasis associated lung adenocarcinoma transcript 1 (non-protein coding)	11q13.1
212296_at	*PSMD14*	Proteasome (prosome, macropain) 26S subunit, non-ATPase, 14	2q14.3
200708_at	*GOT2*	glutamic-oxaloacetic transaminase 2, mitochondrial	16q21
204786_s_at	*IFNAR2*	Interferon (alpha, beta and omega) receptor 2	21q22.1
215543_s_at	*LARGE*	Like-glycosyltransferase	22q12.3

Phosphofructokinase, muscle (*PFKM*) encodes for the enzyme called phosphofructokinase and catalyses the phosphorylation of fructose-6-phosphate to fructose-1,6-bisphosphate (F-1,6BP). F-1,6-BP is broken down into glyceraldehyde 3-phosphate and dihydroxyacetone phosphate under the catalysis of *ALDOA* or *ALDOC* enzymes. In this study *PFKM* is down regulated across all the regions. Several studies have pinpointed the relation between the increased activity of glycolysis and AD [78, 79]. We find *PFKM* under expressed across all the regions. Other researchers have also reported that *PFKM* may be related with the progress of AD in EC region [80], and differential expression of *PFKM* has been studied on different rat brain regions in relation to AD [81]. Brooks et al. [82] reported the down regulation of *ALDOA, ALDOC* and *PFKM* in AD.

Proteasome (Prosome, Macropain) Subunit, Beta Type, 2 (*PSMB2*) encodes for the protein proteasome subunit beta type-2 which is responsible for the degradation cytosolic and nuclear proteins in the cell. Proteasomes are a major part of eukaryotic cells and cleave peptides in an ATP/ubiquitin-dependent process in a non-lysosomal pathway. Hence proteasome plays an inportant role in the ubiquitin-proteasome system, which is an important mechanism in the regulation of cell cycle, differentiation, transcription, signalling, cell growth and death [83]. Several studies have indicated that aberrations and deregulations of ubiquitin proteasome system contribute to the development of neurodegenerative diseases such as AD [84–86]. These studies present indirect evidence for the role of *PSMB2* in the pathology of AD. Moreover, the differential expression and co-regulation of *PSMB2* with *RPL30* in HIP region of mouse brain has been reported in relation to AD [87, 88]. To the best of our knowledge there is no study available to indicate the role of *PSMB2* in relation with AD in humans. Our study shows the down regulation of *PSMB2* across all the regions in relation with AD. These results taken together point to further studies on *PSMB2* may lead to new insights about AD development.

WNK Lysine Deficient Protein Kinase 1 (*WNK1*) encodes for cytoplasmic serine-threonine kinase which plays a key role in the regulation of blood pressure by controlling the transport of sodium and chloride ions. There is no evidence available for the relation of *WNK1* and AD progression. However, there is evidence of MAPK/ERK signalling pathway activation by *WNK1* via the stimulation of the epidermal growth factor (*EGF*) [89–91]. As mentioned before, *MAPK/ERK* signalling is highly associated with the pathogenesis of AD [92, 93]. In 2008, Shekarabi et al. [94] reported that mutations in the nervous system resulting from the over expression of *WNK1* cause neurodegerative disorder called hereditary sensory neuropathy type II. More recently, the over expression of *WNK1* was reported to be associated with schizophrenia, a neurodevelopmental disorder [95]. In our study, *WNK1* is over expressed across all the regions and it is worth noticing that *MAPK* signalling is one of the pathway which is dysregulated in all the five regions in our study.

Ribosomal Protein L15 (*RPL15*) encodes for the protein 60S ribosomal L15 which plays a key role in RNA binding. Up-regulation of *RPL15* is reported in relation with AD in the HIP region of brain [96]. Our study also reports the up-regulation of *RPL15* in the pathogenesis of AD, not only related with the HIP region, but also in EC, MTG, PC and SFG. It has also been reported that *RPL15* is closely related with parkinson’s disease [97] and other brain disorders [98, 99]. Our result along with these studies shows that *RPL15* may be a good reference gene for AD pathogenesis across different brain regions.

Prothymosin, Alpha (*PTMA*) works as a mediator for immune function by conferring resistance to certain opportunistic infections like Candidiasis and Kaposi’s Sarcoma. Recent studies have reported the over expression of *PTMA* in AD [100, 101] and the role in *TGF* *α* induced apoptosis and estrogen receptor *α* induced proliferation [102]. Our study also reports the up regulation of *PTMA* associated with AD in EC, HIP, MTG, PC and SFG.

Sema Domain, Immunoglobulin Domain (Ig), Transmembrane Domain (TM) And Short Cytoplasmic Domain, (Semaphorin) 4C (*SEMA4C*) encodes for the protein Semaphorin-4C which is essential for the activation of p38 MAPK. The importance of SEMA4C in the nervous system is well defined as it plays an important role in the development and plasticity of central nervous system [103–105]. Our study shows the over expression of *SEMA4C* across all the regions. The expression of *SEMA4C* was originally identified in the nervous system and widely expressed in the brain of embryonic and neonatal mouse [106]. p38 MAPK is emerging as a new AD treatment strategy, and the dysregulation of p38 MAPK in AD is well defined and studied [92, 93, 107]. There is no single study that shows the relation of *SEMA4C* associated with AD.

Gephyrin (*GPHN*) encodes for a neuronal assembly protein that activates the inhibitory neurotransmitter receptors. The reduced expression of *GPHN* and synaptic dysfunction has been reported in relation to Alzheimer’s disease [108], and plaque-like accumulations of gephyrin in AD was identified by Hales et al. [109]. We also confirm the down regulation of *GPHN* across all the brain region studied here.

Member of the RAS Oncogene Family (*RAB2A*) encodes for the Rab family protein which is involved in GTP binding, hydrolysis and participates in cell cycle. Only a handful of studies have been performed for *RAB2A* related with neurodegenerative disorders. In 2014, the dysregulation of *RAB2A* in HIP region was reported by Parra-Damas et al. [110]. We also confirm the down regulation of *RAB2A* in AD samples across EC, HIP, MTG, PC and SFG.

RWD Domain Containing 2A (*RWDD2A*) is a conserved region of about 110 amino acid residues. It can be found in many ring finger proteins, dead like helicases and WD repeat containing proteins and is mainly involved in protein interaction. A recent age-related study using a mouse model of AD has reported over expression of *RWDD2A* in both HIP and Cortex regions [111]. To the best of our knowledge this is the first study that reports the over expression of *RWDD2A* in five different human brain regions.

Gamma-Aminobutyric Acid (*GABA*) B Receptor, 1 (*GABBR1*) is the main inhibitory neurotransmitter in the human central nervous system. GABBR1 uses the ionotropic receptors to produce fast synaptic inhibition and metabotropic receptors to produce slow and prolonged inhibitory signals. This gene also plays a key role in hippocampal long-term potentiation, slow wave sleep, muscle relaxation and antinociception.*GABBR1* is encoded for the Major Histocompatibility Complex (MHC) [112] and the association between MHC and AD has been reported in the literature [113]. The expression of *GABBR1* is widely studied in relation with brain disorders [114–116]. In 2005, Iwakiri et al. [117] reported that this gene could contribute to the AD pathology in the HIP region through the alternations in the balance between the neurotransmitter systems. We report the under expression of *GABBR1* in AD across five different brain regions. Detailed study of this gene can contribute new insights to the disease progression as this gene is one of the main transmitters in the nervous system.

Proteasome (Prosome, Macropain) 26S Subunit, Non-ATPase, 14 (*PSMD14/RPN11*) is a multi protein complex that plays an important role in the degradation of ubiquitinated intracellular proteins. In our study, *PSMD14* is under expressed across all the brain regions. Recently, the down regulation of *PSMD14* has been reported in relation with AD [118] and other brain disorders [119, 120].

Glutamic-Oxaloacetic Transaminase 2, Mitochondrial (*GOT2*) is a pyridoxal phosphate-dependent gene that plays an important role in amino acid metabolism. Several studies have reported the down regulation of GOT2 in AD [121, 122], which is consistent to our results in all five regions. A recent study of RNA transcripts performed by our group also reported that *GOT2* may play a key role in AD pathology [123].

Interferon (Alpha, Beta And Omega) Receptor 2 (*IFNAR2*) encodes for the type I membrane protein which is involved in the binding and activation of the receptor that stimulates Janus protein kinases like *STAT1* and *STAT2*. Our study indicates the over expression of *IFNAR2* acroos five different brain regions. Several other studies have also reported the up regulation of *INFAR2* in AD pathology [124–126].

Like-Glycosyltransferase (*LARGE*), one of the largest genes in the human genome, encodes for the protein glycosyltransferase, which participates in glycosylation of alpha-dystroglycan and the synthesis of glycoproteins. Glycosylation is closely associated with AD and other neurodegenerative disorders [127–129]. In this study, *LARGE* is under expressed in different brain regions. Studies shows that *LARGE* plays key role in the glycosylation [130]. Studies also shows that *LARGE* is closely associated with other brain disorders like Neuronal Migration Disorder, dystroglycanopathies and Muscle–eye–brain disease [131, 132].

Fibroblast Growth Factor (Acidic) Intracellular Binding Protein (*FIBP/FGF*) encodes for an intracellular protein that binds selectively to acidic fibroblast growth factor (aFGF). In our study *FIBP/FGF* is up-regulated across all the regions. Many other studies also reported the dysregulation of *FIBP/FGF* in relation with AD [133–135].

Gamma-Aminobutyric Acid (*GABA*) A Receptor, Gamma 2 (*GABRG2*) is the major inhibitory neurotransmitter in the mammalian brain, where it acts as ligand-gated chloride channels. Several studies have shown the down regulation of *GABRG2* in HIP region of the brain in relation with AD and other neurodegenarative disorders [136–138]. Our study also indicate the down regulation of *GABRG2*, not only in HIP region but also in EC, MTG, PC and SFG.

We also find 23 non coding features that are differentially expressed across all the brain regions. Although non coding RNAs are the least understood, we cannot disregard their potential for functionality. So it is worth noting the importance of non coding features in AD as it may act as strong future candidates for diagnostic and therapeutic tools in the clinical treatment of AD. The pathway analysis of these non coding features resulted with 13 pathways, given in Table 3.

The expression of non-coding features has already been linked to several human diseases such as cancer and neurological disorder. Recently, studies of neural differentiation have reported that the non-coding features act as additional players in the development of neurological disorders [139–141]. In the list of 23 non-coding features, *MALAT1, SNORD14E* and *NEAT1* are already studied and reported to be related with neurodegenerative disorders. Our study shows the under expression of *MALAT1, SNORD14E* and the over expression of *NEAT1* across five different brain regions. Very recently, studies reported that heat-stress–related genes like *SNORD14E* is associated with the neurodegenerative disorders such as AD, Parkinson’s disease and Huntington disease [142, 143]. The key role of *NEAT1* has been reported in relation to neuronal activity, growth and branching [141, 144]. Also the up-regulation of *NEAT1* is reported in Huntington’s disease [145–147]. To the best of our knowledge, this is the first study that report the over expression of the *NEAT1* in relation with AD. Other non-coding feature that we find overexpressed, *RP11-488L18.10*, has been reported as differentially expressed in AD samples astrocyte cells [17].

Among these non coding features, four are microRNA precursors over expressed across five brain regions. MicroRNAs play a key role in the development and function of nervous system, as 70% of known microRNA precursors are expressed in the brain [148, 149]. These microRNA precursors are dynamically regulated during brain development and target different genes and perform different functions in the brain. A study by Sempere et al. [150] reported a group of 17 microRNAs, including hsa-mir-7-1 (miR-7), playing a key role in neuronal differentiation, maturation, and/or survival in human. miR-7 controls the epidermal growth factor receptor (*EGFR*) related signalling and promotes cell differentiation [151, 152]. The role of miR-7 in modulating *α*-synuclein levels in the nervous system has also been reported in relation to AD [153] and parkinson’s disease [154]. Several studies have shown the involvement of miR-7 with brain development and diseases [154, 155]. Moreover, *EGFR* and the related MAPK signalling pathway is in the top list of our pathway analysis result. That shows that miR-7 may have an important role in the development of AD, even though further studies are needed to characterise its role. hsa-mir-570 (miR-570) has already been reported in relation with brain aging and neurodegeneration [156].

hsa-mir-1229 (miR-1229) has been detected as a suitable biomarker for colon cancers [157]. To the best of our knowledge the role of miR-1229 has not been studied in relation with AD before. Differential expression of miR-1229 across five different brain regions is a novel result from this study, as is the differential expression of the relatively unknown microRNA hsa-mir-6821. Even though the mechanism behind the role of miRNAs in disease development remains controversial, our findings suggest a possible suppression of various cellular functions through the differential expression of this group of miRNAs. Among them, miR-7 and miR-570 have been the subject of intense study in relation to AD. From the miRDB-reported gene targets [158] of miR-7, 23 genes are in our Coloured (α,β)-*k* Feature Set result, while the targets of miR-570 in our result list are 88. Among the top 15 genes given in Table 5, *RPL15* is targeted by miR-7 and *WNK1* is targeted by miR-570. Although the understanding of the function and role of non-coding features in different diseases lags far behind that of their related protein partners, these features will have particular significance in the future as the role of non-coding features in relation with neurodegenerative disorders becomes increasingly recognised.

The comparison with the list of genes collected from The Human Protein Atlas shows that 328 genes that are normally expressed in human brain appear either up or down regulated in AD with respect to normal brain tissue in our results. An additional 17 genes which are not expressed in normal human brain appear over expressed in AD. The top 3 genes with good BF-Value are explained here.

BEN Domain Containing 5 (*BEND5*) plays an important role in the preservation of the nervous system integrity by controlling the passage of harmful substances and inflammatory cells into the brain. Our study shows the up-regulation of BEND5 in AD. Other studies also reported the up-regulation of *BEND5* in relation with AD [159, 160].

Zinc Finger Protein 415 (*ZNF415*) plays an important role in the gene expression pathway. Our study shows the over expression of this gene across five different brain regions. Other studies also shows the differential expression of *ZNF415* related to AD [161].

TSPY-Like 5 (*TSPYL5*) plays an important role in cell growth and cellular response to gamma radiation via regulation of the Akt signaling pathway. Our study shows the up-regulation of this gene across different brain regions. The up-regulation of *TSPYL5* has been reported to be associated with AD [162].

Finally, this combined study of AD datasets provided us with new candidate genes that are consistently differentially expressed across five different brain regions. Further investigation on *PSMB2, WNK1, RPL15, SEMA4C, RWDD2A* and *LARGE* may provide us new insights to the development of AD. Hopefully, more research on miR-7 and miR570 may contribute more to the Ad pathology. We also showed that there are significant differences in the gene expression levels in different brain regions, suggesting there are unique regional activity patterns of AD affected brain regions and significant differences in the neurodegenerative mechanisms within each region.

Collectively these results illuminate the potential of these genes to provide insights into AD pathogenesis and initial hope that microRNA precursors may serve as useful biomarkers for AD severity even if further study needed. It is clear that researchers can benefit from these highly AD correlated genes that may serves as markers for AD.

## Conclusion

A meta-analysis methodology with a clear mathematical interpretation and guarantees leads to a largely improved set of markers of AD. We uncover a set of six genes and two miRNAs that warrant further investigation for their high significance in AD-related processes. While development of drugs directed to treatment of AD still lags behind, these new findings may provide new insights into the disease mechanisms and thus make a significant contribution in the near future towards understanding, prevention and cure.

## Supporting Information

S1 Fig Full-sized version of Fig 1. (TIFF)

S2 Fig Full-sized version of Fig 2. (TIFF)

S1 Table Sample details. The details of samples for each brain region, and the individual identification across regions based on related meta-data.
(XLS)

S2 Table Individual Region (α,β)-*k* Signatures. The Individual (α,β)-*k* analysis signature result for EC, HIP, MTG, PC, SFG and VCX region. For each signature, the features with Bonferroni corrected p-value < 0.0001 are highlighted.
(XLS)

S3 Table Extended Individual Region Pathway Analysis. The pathways resulting from the EASE annotation of complete individual region (α,β)-*k* signatures for EC, HIP, MTG, PC, SFG and VCX regions.
(XLS)

S4 Table Most Significant Individual Region Pathway Analysis. The pathways resulting from the EASE annotation up to EASE score < 0.06, for the highlighted parts of the individual region (α,β)-*k* signatures in S2 Table.
(XLS)

S5 Table Individual Region Signature Heatmaps. Heatmaps for the individual analysis resulting probes that have a BF-value < 0.0001, highlighted in S2 Table.
(XLS)

S6 Table MicroRNAs and Non-coding Features. For each individual region, a summary of microRNAs present in the (α,β)-*k* signature list is presented. For the integrated result signature from the Coloured (α,β)-*k* method, a list of microRNAs and other non-coding features is given.
(XLS)

S7 Table Coloured (α,β)-*k* and Generalised (α,β)-*k* Signature Results. Results for the integration of data from five regions, integrating only by regions (Coloured (α,β)-*k*) and by region and shared samples (Generalised (α,β)-*k*). The common probes between these two approaches are marked. The EASE annotated pathways are also given in this file.
(XLS)

S8 Table Comparison with two Meta-Analysis Methods. The result of the combined meta-analysis using two methods (RankProd and GeneMeta) compared to the results of the Coloured (α,β)-*k* and Generalised (α,β)-*k* approaches. For RankProd results, the up or down regulated RankProd ranksum is also given for Coloured (α,β)-*k* and Generalised (α,β)-*k* signature probes, For GeneMeta results, the absolute value of the two-sided z-score and the two-sided FDR is given for Coloured (α,β)-*k* and Generalised (α,β)-*k* signature probes. In all cases, results are sorted in descending order of importance.
(XLS)

S9 Table Result of the Sensitivity Analysis. Sensitivity of the Coloured (α,β)-*k* results to the suppression of one or two region’s data. In the table, common probes between the results of Coloured (α,β)-*k* for the five regions, and when the EC, the HIP or both MTG+PC regions are excluded from the data.
(XLS)
