# Supplementary figures and images for: Identification of Differentially Expressed Genes through Integrated Study of Alzheimer’s Disease Affected Brain Regions
Source: PLoS One. 2016 Apr 6;11(4):e0152342. doi: 10.1371/journal.pone.0152342 (PMC4822961; doi:10.1371/journal.pone.0152342)

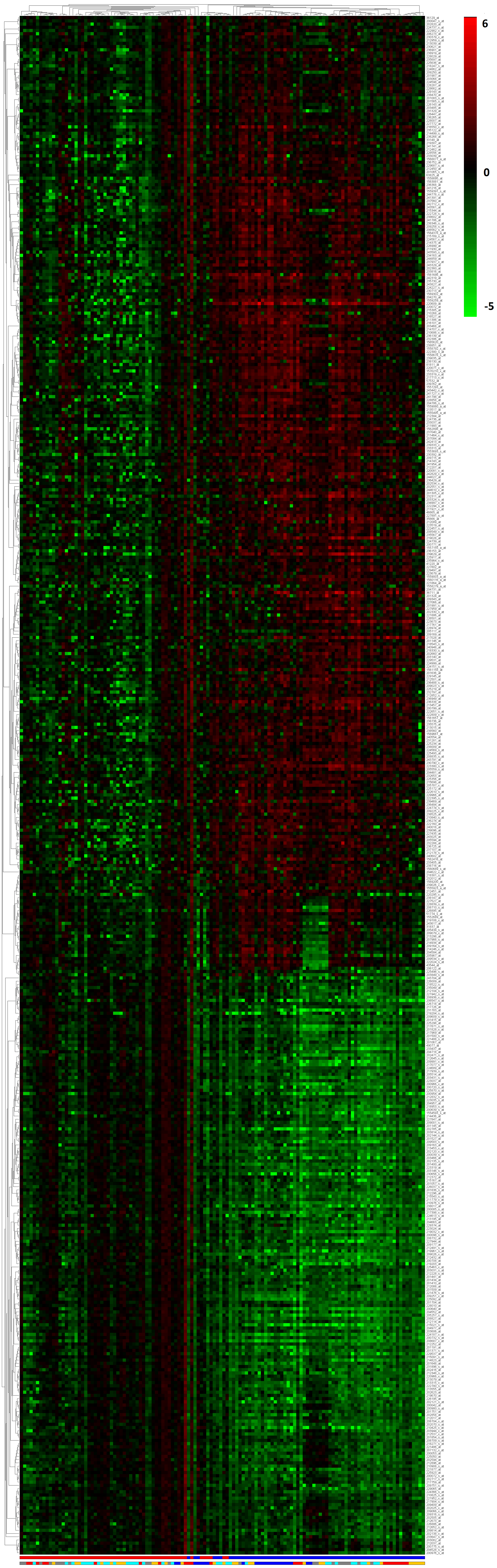

Supplement: S1 Fig — (TIFF) [file pone.0152342.s001.tiff]

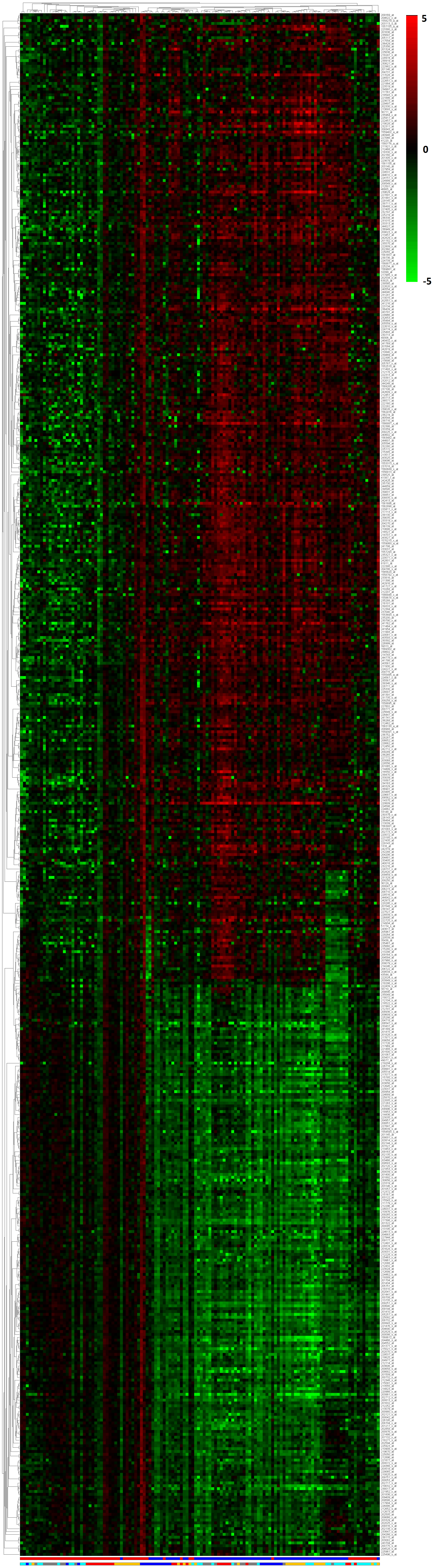

Supplement: S2 Fig — (TIFF) [file pone.0152342.s002.tiff]
